# P66Shc is increased in peripheral blood mononuclear cells of the patients with obstructive sleep apnea

**DOI:** 10.7150/ijms.80343

**Published:** 2023-02-13

**Authors:** Xing Lyu, Jingyao Cai, Rongxuan Yan, Peiying Huang, Hui Gong, Jieting Peng, Yang Liu, Shizhen Li, Shengyu Tan, Min Hu, Xiangyu Zhang

**Affiliations:** 1 Department of Clinical laboratory Medicine, The Second Xiangya Hospital, Central South University, Changsha, Hunan 410011, China; 2 Department of Otolaryngology-Head and Neck Surgery, The Second Xiangya Hospital, Central South University, Changsha, Hunan 410011, China; 3 Department of Geriatrics, The Second Xiangya Hospital, Central South University, Changsha, Hunan 410011, China; 4 Hunan Clinical Medical Research Center for Geriatric Syndrome, Changsha, Hunan 410011, China

**Keywords:** p66Shc, peripheral blood monocytes, obstructive sleep apnea, oxidative stress

## Abstract

**Objective:** Obstructive sleep apnea (OSA) is characterized by nocturnal intermittent hypoxemia and linked to oxidative stress. Evidence demonstrated that p66Shc plays a key role in regulating oxidative stress. This study aimed to investigate the expression of p66Shc in peripheral blood mononuclear cells (PBMCs) of patients with OSA and the association with polysomnographic parameters.

**Methods:** Fifty-four OSA subjects and 19 no OSA controls were enrolled in this study. All the subjects underwent standard polysomnography. P66Shc mRNA and protein levels in the PBMCs were detected by quantitative real-time polymerase chain reaction and western blotting. Plasma 3-nitrotyrosine (3-NT), oxidized low density lipoprotein (oxLDL), and advanced oxidation protein products (AOPP) were measured by ELISA method.

**Results:** P66Shc mRNA and protein levels in PBMCs were significantly higher in OSA patients than in controls. P66Shc mRNA was positively correlated with plasma 3-NT, oxLDL, AOPP, hypopnea index (AHI), oxygen desaturation index (ODI), percentage of total sleep time with oxygen saturation (SaO_2_) below 90% (CT90), epworth sleepiness scale (ESS) and lymphocytes; negatively correlated with lowest SaO_2_ (LSaO_2_) and mean SaO_2_ (MSaO_2_). Further multivariate linear regression analysis showed that p66Shc mRNA levels were independently associated with AHI, MSaO_2_ and CT90.

**Conclusions:** Oxidative stress regulator p66Shc may play a role in the pathophysiology of OSA and might serve as a potential biomarker for this disease.

## Introduction

Obstructive sleep apnea (OSA) is the main cause of sleep disorders with a high prevalence ranged from 9 to 38% in the general population^1^, ^2^. The incidence rate increases with age and is approximately 3-10% at the age of 30-49, 9-17% at 50-70^3^, ^4^. It is characterized by repetitive episodes of upper airway collapse during sleep which may result in increased sympathetic activity, intermittent hypoxia and hypercapnia 5. Evidence has indicated that OSA related cardiovascular disease and morbidity are in-creasing worldwide due to the improvement of living conditions and increased rate of obesity 6.

The pathophysiological mechanisms of OSA are still not utterly defined. Intermittent hypoxia/reoxygenation episodes activated oxidative stress and reactive oxygen species (ROS) generation play a role in this process^7^, ^8^. Recently, accumulating evidences demonstrate that p66Shc is an important regulator of oxidative stress 9. p66Shc belongs to the SHC family of adaptor proteins, which includes p46Shc, p52Shc and p66Shc three isoforms 10. P66Shc is the only isoform that includes an exclusive redox functional domain. Due to the existence of this domain, p66Shc participates in the regulation of mitochondrial ROS generation and oxidative stress in multiple cells and tissues 11. P66Shc has also been shown to mediate endothelial dysfunction. More than three decades of active research indicates that the most robust effects of p66Shc is regulating vascular endothelial functions in a broad range of pathological conditions including diabetes and coronary artery disease (CAD)^12^, ^13^. Studies had reported that p66Shc mRNA levels were increased in the peripheral blood mononuclear cells or monocytes (PBMCs) of the patients with diabetes mellitus or CAD^14^-^16^. The role of p66Shc in the OSA patients has not been investigated. Whether p66Shc is involved in the physiological process of OSA and its correlation with the severity of intermittent hypoxia remain unknown. Thus, p66Shc mRNA and protein expression in PBMCs of OSA patients and controls were detected and the relationship between the p66Shc mRNA expression and polysomnographic parameters, oxidative stress markers was analyzed in this study.

## Materials and Methods

### Human Subjects

The study recruited 73 consecutive inpatients or outpatients in the Second Xiangya Hospital of Central South University. All the subjects underwent standard polysomnography (PSG) due to the clinical suspicion of OSA. The results of the Epworth Sleepiness Scale (ESS) were recorded. A score of >10 on the ESS denoted extreme daytime drowsiness. OSA was categorized as AHI≥5 events/h based on the pertinent clinical practice recommendations by American Academy of Sleep Medicine (AASM). OSA was further divided into mild (5≤AHI<15 events/h), moderate (15≤AHI<30 events/h), and severe OSA (AHI≥30 events/h). In this study, 54 were diagnosed with OSA, 19 without OSA. Among the OSA patients, 12 had mild, 15 had moderate, and 27 had severe OSA.

Clinical information, including age, gender, height, weight, systolic blood pressure (SBP), and diastolic blood pressure (DBP), was collected. BMI (kg/m^2^) was determined by dividing body weight (kg) with the square of body height (m^2^). Complete blood cell count (CBC), glucose, lipids, liver and kidney function index were tested in the Department of the Clinic Laboratory at The Second Xiangya Hospital.

Subjects with following diseases or conditions were excluded in this study: (1) other types of sleep disorder or received continuous positive airway pressure treatment; (2) chronic obstructive pulmonary disease and asthma; (3) cardiovascular and cerebrovascular diseases; (4) diabetes mellitus; (5) acute infection; (6) history of or active cancer; (7) abnormal hepatic or renal function; (8) autoimmune diseases; (9) cognition impairment, psychiatric disorders, or poor cooperation; (10) total sleep time below 4 hours during PSG examination; and (11) pregnancy.

The study was approved by the institutional ethics committee of the Second Xiangya Hospital of Central South University. All participants signed the informed consent form. All experiments were conducted in conformity with the applicable standards and regulations.

### Polysomnography

Electroencephalogram, electrooculogram, electromyogram, electrocardiogram, SaO_2_, oral and nasal airflow were recorded. The sleep stage was scored using AASM criteria. Apnea was defined as the total stoppage of airflow lasting at least 10 seconds. Hypopnea was defined as a 30% decrease in airflow signal amplitude for at least 10 seconds, followed by a 3% decrease in SaO_2_. AHI was determined by averaging the number of apneas and hypopneas during each hour of sleep time. The LSaO_2_ and MSaO_2_ parameters were also recorded as indices of nocturnal hypoxemia.

### Plasma Samples Collection and PBMCs Isolation

Blood specimens were obtained from the antecubital inferior caval vein of the subjects in a fasting state using EDTA anticoagulant tube. PBMCs were extracted using Ficoll-Paque density gradient centrifugation and the conventional density gradient separation procedure (TBD Science, Catalog#HY2015, China). Isolated PBMCs were washed with 1×PBS three times and counted with a Neubauer chamber. Then, 1.5×10^6^ PBMCs were resuspended in 600µl RLT buffer (catalog#TR118, Molecular Research Center, USA) according to the customer's specifications and frozen at -80°C for further analysis.

### Enzyme-linked Immunosorbent Assay

The level of plasma 3-NT (Elabscience, Catalog#E-EL-0040c, China), oxLDL (Elabscience, Catalog#E-EL-H6021, China) and AOPP (Abbkine, Catalog#KTB1060, China) were detected with the enzyme-linked immunosorbent assay (ELISA) commercial kits according to the recommendations of the manufacturers.

### Quantitative Real-Time Polymerase Chain Reaction

RNA was extracted with UNIQ-10 Column Trizol Total RNA Isolation Kit (Sangon Biotech, Catalog#B511321-0100, China) and transcribed reversely to cDNA using GoScript^TM^ Reverse Transcription System (Promega, Catalog#A5001, USA). RT-qPCR was used to detect mRNA levels using the Roche Light Cycler 96 system (Roche, Switzerland) and GoTaq^®^ qPCR Master Mix (Promega, Catalog#A6002, USA) based on the manufacturer's instruction. The amplify sequences used in this experiment were as follows: P66Shc forward: 5'-TGAGG GTGTG GTTCG GACTA AGG-3', reverse: 5'-CCGCA GAGAT GATGG GCAAG TG-3'; β-actin forward: 5'-TCGTG CGTGA CATTA AGGAG-3', reverse: 5'-GATGT CCACG TCACA CTTCA-3'. The relative expression levels of p66Shc mRNA were calculated using the 2^-ΔΔCt^ method normalized to β-actin.

### Western Blot

Total cell lysates were prepared from PBMCs and washed repeatedly in cold PBS before being transferred to an ice-cold RIPA buffer (Millipore, Catalog#89900, USA). Protein concentration was determined using the BCA protein assay kit (Thermo Fisher, Catalog#23235, USA). Equivalent amounts (10µg per well) of protein sample were separated by 10% SDS-PAGE (Beyotime Institute of Biotechnology, Catalog# P0012A, China) and transferred to a PVDF membrane (Millipore, Catalog#IPVH00010, USA). Immunoblots were imaged using an Amersham Biosciences 600 imager. The protein bands were scanned and analyzed by Image J software. Antibodies used in this study were: SHC (1:1000, BD Biosciences, Catalog#610878, USA), β-actin (1:50000, Cell Signaling Technology, Catalog#4970T, USA).

### Statistical Analysis

The traits of the patients had been expressed as ± standard deviation (SD), median (interquartile range, IQR), or number (proportion). The ANOVA is used to examine continuous variables among groups, and the Student's t-test was employed to determine the distinction between two groups. The Wilcoxon rank sum test was utilized for non-parametric data. Pearson correlation coefficient and multivariate linear regression model were used to examine relationships between continuous variables. Statistical significance was described as a p-value<0.05 (within a 95% confidence interval). The SPSS software program 25.0 was used for the statistical analysis.

## Results

### Basic Characteristics

A total of 73 subjects were included in this study. The baseline characteristics of the no OSA control group (n=19) and the OSA group (n=54) were shown in Table 1. There were no significant differences in age, sex, SBP, DBP, number of current smokers and drinkers between the two groups. BMI (*p*=0.008), AHI (*p*<0.001), CT90 (*p*<0.001), ODI (*p*<0.001) and ESS (*p*<0.001) were considerably greater in the OSA group than in the control group. LSaO_2_ (*p*=0.003) and MSaO_2_ (*p*<0.001) were lower in OSA group than in the control group. The white blood cells count (*p*=0.049), lymphocytes count (*p*=0.006), monocytes count (*p*=0.024), triglyceride (*p*=0.027), ALT (*p*=0.039), AST (*p*=0.025) were significantly higher in the OSA patients than in the controls. There were no significant differences in other biological parameters between the two groups.

Further analysis in the OSA patients of different extent and the controls showed that there were no significant differences among the four groups in age, sex, SBP, DBP, number of current smokers and drinkers. Compared with the control group, higher BMI was observed in the severe OSA group. AHI, CT90, ODI, ESS were significantly higher and LSaO_2_ and MSaO_2_ were significantly lower in the severe OSA group than in the other three groups (Table 2).

### Association of oxidative stress markers with p66Shc in the subjects

The plasma oxidative stress biomarker 3-NT, oxLDL, AOPP and mRNA levels of p66Shc in PBMCs were detected and the results showed their levels were significantly higher in the OSA group than in the control group (Table 1). When further compared among OSA patients of different extent and the control group, the levels of 3-NT, oxLDL, AOPP and p66Shc mRNA gradually increased with the severity of OSA; 3-NT, AOPP and p66Shc mRNA levels were significantly higher in the severe OSA group than in the other three groups; and oxLDL levels were significantly higher in the severe OSA group than in the control and mild OSA groups (Table 2).

**Figure 1:**
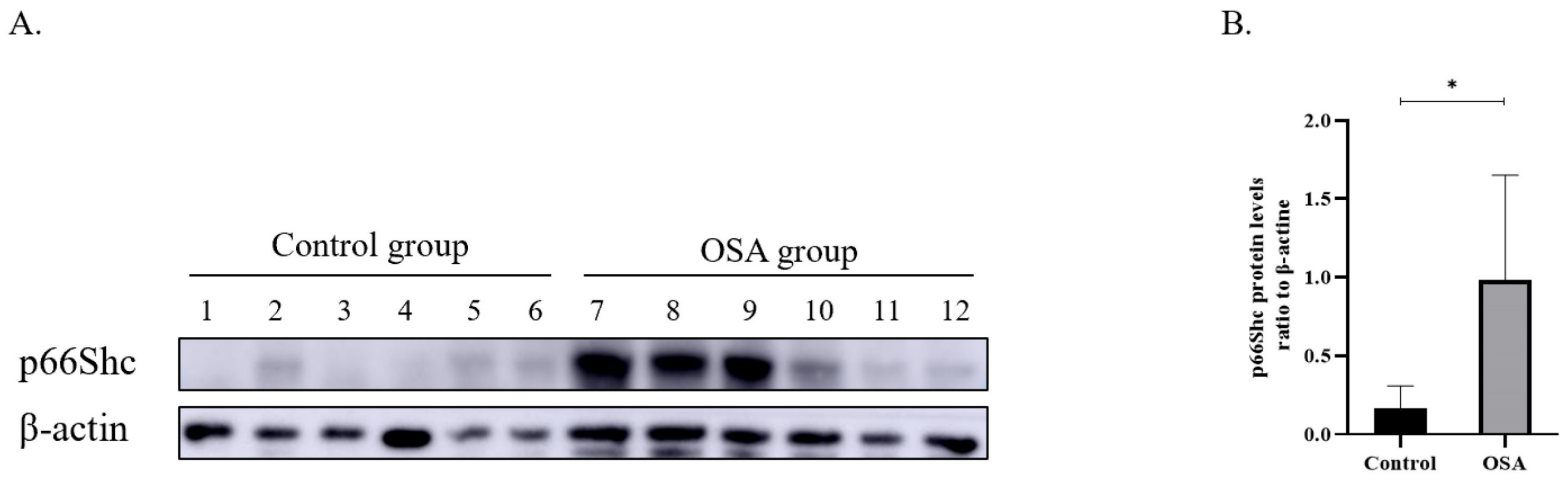
** (A)** P66Shc Protein expression in the peripheral blood monocytes from controls (n=6) and severe OSA patients (n=6) by Western blot. β-actin was used as a loading control. **(B)** Densitometric analysis of the Western blot in A. *p<0.05

Six no OSA controls and 6 severe OSA sufferers were chosen randomly and p66Shc protein in PBMCs were detected. The results displayed that the severe OSA sufferers had greater p66Shc protein expression than the controls (Figure 1).

**Figure 2:**
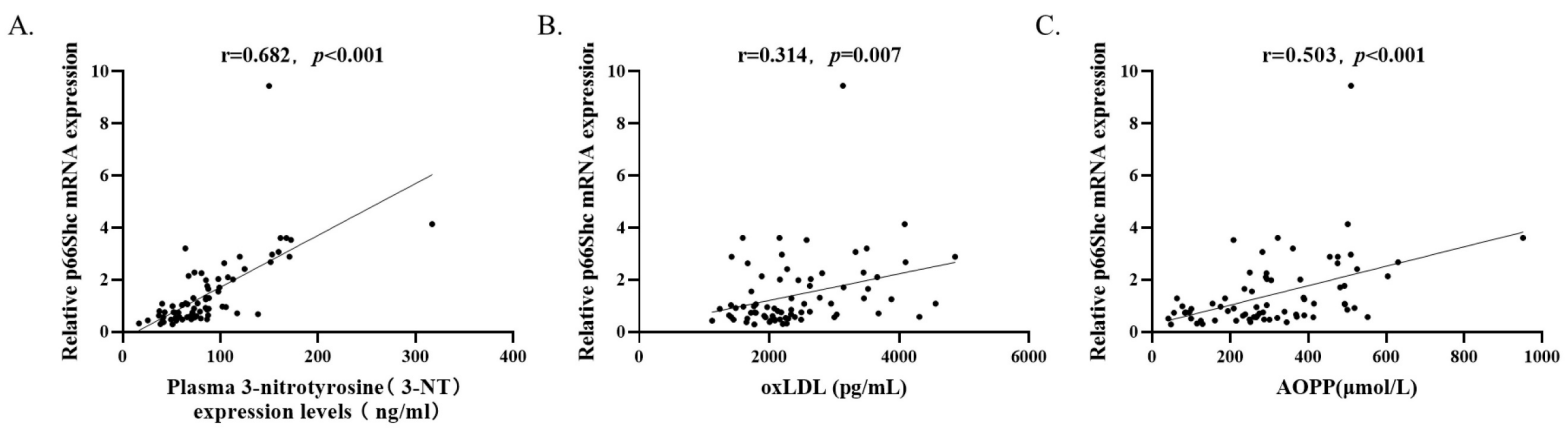
P66Shc mRNA levels (n=73) were positively correlated with **(A)** plasma 3-NT, **(B)** oxLDL, and **(C)** AOPP levels by Spearman's rank correlation coefficient.

By analyzing all the subjects (n=73) with Spearman's rank correlation coefficient, it is observed that p66Shc mRNA levels were positively correlated with plasma 3-NT levels (r=0.682, *p*<0.001), oxLDL levels (r=0.314, *p*=0.007), and AOPP levels (r=0.503, *p*<0.001) (Figure 2).

### The correlation of p66Shc mRNA levels in the PBMCs with sleep and clinical parameters

**Figure 3:**
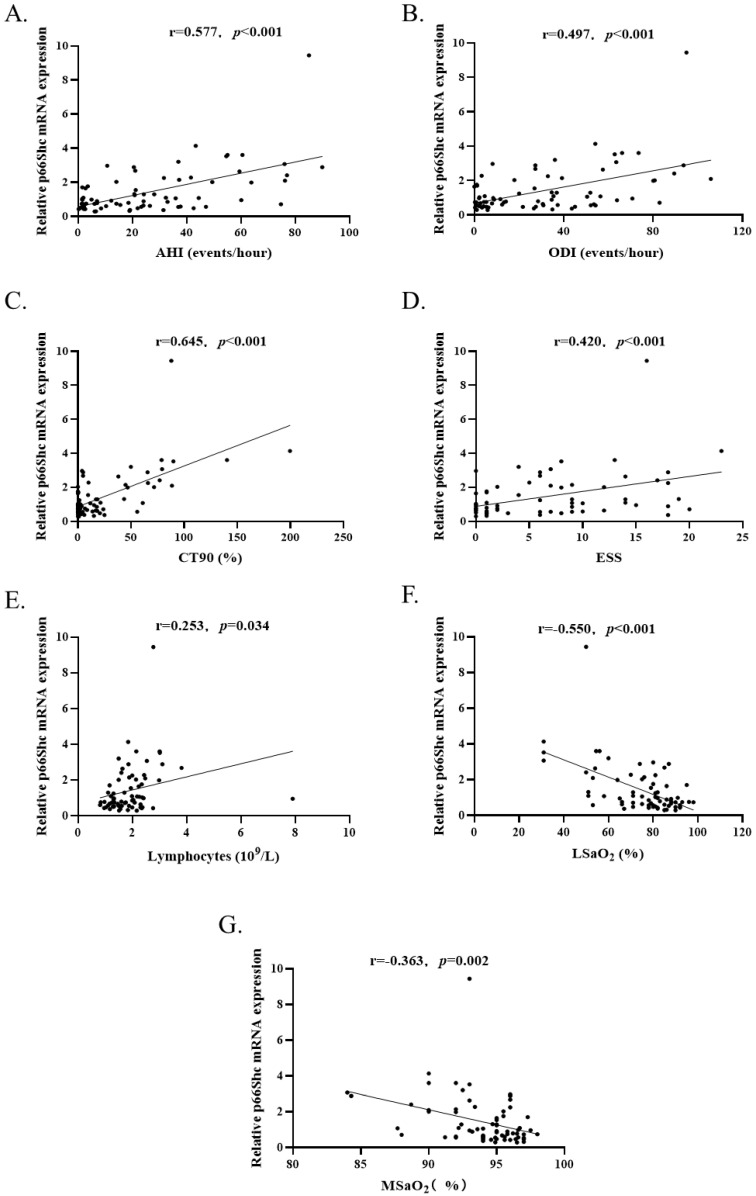
** (A-E).** P66Shc mRNA levels (n=73) were positively correlated with AHI, ODI, CT90, ESS and blood lymphocytes count. **(F** and **G)** P66Shc mRNA levels were negatively correlated with LSaO_2_ and MSaO_2_.

To investigate whether the levels of p66Shc mRNA were correlated with sleep and clinical parameters, Spearman's correlation was used to analyzing the total study subjects (n=73). The results revealed that p66Shc mRNA levels were positively correlated with AHI (r=0.577, *p*<0.001), ODI (r=0.497, *p*<0.001), CT90 (r=0.645, *p*<0.001), ESS (r=0.420, *p*<0.001) and lymphocytes (r=0.253, *p*=0.034), negatively correlated with LSaO_2_ (r=-0.550, *p*<0.001) and MSaO_2_ (r=-0.363, *p*=0.002) (Figure 3). There was no correlation between p66Shc mRNA and age, sex, BMI, SBP, DBP, WBC, MNC, lipids, the indices of liver and renal functions.

Univariate and multivariate regression analyses were conducted to further investigate the relationship between p66Shc mRNA levels and the clinical variables. In univariate analysis, AHI, LSaO_2_, MSaO_2_, CT90, ODI, ESS, and LYM were associated with p66Shc mRNA levels (Table 3). These seven variables as well as age, sex, and BMI were tested in a multivariate model. Only AHI (Beta: 0.067, 95%CI: 0.031 to 0.104; *p*<0.001), MSaO2 (Beta: 0.264, 95%CI: 0.100 to 0.428; *p*=0.002) and CT90 (Beta: 0.020, 95%CI: 0.008 to 0.032; p=0.002) were independently associated with p66Shc mRNA levels (Table 3).

## Discussion

Oxidative stress is considered as the underlying mechanism in the pathogenesis of OSA 17. 3-NT is produced as a result of the reaction of nitric oxide with other radicals, which is associated with many diseases 18. oxLDL is a marker of lipid peroxidation and oxidative stress. Studies have found that OSA patients have higher plasma 3-NT and oxLDL levels than healthy individuals^19^-^21^. AOPP is a marker of both oxidative stress and inflammation 22. A study showed that AOPP concentrations in saliva samples are higher in the morning than in the evening in patients with OSA 23. Moreover, Yağmur et al., found that the plasma AOPP levels had a positive correlation with AHI and ODI 24. Our results similar to previous studies and found that 3-NT, oxLDL and AOPP were increase in OSA patients, which reconfirmed that OSA is associated with oxidative stress.

OSA was linked to increased intracellular ROS levels produced by certain monocyte and granulocyte subpopulations 20. Previous research has shown an expanded manufacturing of ROS in leukocytes in OSA sufferers 25-27. Thus, CBC was analyzed in the current study and found that patients with severe OSA had a significantly higher lymphocytes and monocytes compared to controls. This demonstrated the possibility that PBMCs may associate with the pathogenesis of OSA.

P66Shc, a member of the ShcA protein family, is critical in the cell response to oxidative stress and elicits the formation of mitochondrial ROS 28. P66Shc is an adaptor protein that contains an additional amino-terminal proline-rich region named CH2, which functions as a redox enzyme implicated in the generation of mitochondrial ROS and translation of oxidative signals 29. *In vitro* and *in vivo* researches have illustrated that p66Shc activity is linked to vascular atherosclerosis and endothelial dysfunction, which rae associated with oxidative stress 30. The level of p66Shc mRNA in PBMCs of patients with diabetes 31 or CAD 15 is notably higher than in healthy individuals.

We first investigate the expression of p66Shc level in PBMCs in patients with OSA and no OSA controls. Our results revealed that p66Shc mRNA and protein levels in PBMCs were considerably increased in the patients with OSA when compared with the controls and positively correlated with 3-NT, oxLDL and AOPP. Furthermore, the three oxidative stress markers and p66Shc mRNA elevated with the severity of OSA; the concentrations were highest in the severe OSA group. In multivariate linear regression analyses, AHI, MSaO_2_ and CT90 were independently associated with p66Shc mRNA levels. These demonstrate that OSA is related to oxidative stress and p66Shc level in PBMCs can reflect the severity of OSA to some extent, and thus p66Shc might serve as a potential biomarker for OSA.

Previous study suggested that genetic ablation of p66Shc could reduce the superoxide generation in PBMCs 32. This indicated that p66Shc expression influenced PBMC function 33, and p66Shc had a role in the modulation of oxidative stress generated by PBMCs. Preceding research have proven that p66Shc is linked with oxidative stress in lymphocytes^34^-^36^. Our results showed that p66Shc levels are only associated with lymphocytes, but not monocytes, white blood cells, which suggested that in OSA patients, p66Shc may be involved in the regulation of oxidative stress in lymphocytes.

It has been reported that p66Shc was also correlated with age, obesity and diabetes. Ciciliot et al. pointed out that p66Shc mRNA and protein levels in human visceral adipose tissue were positively correlated with BMI.^37^ According to Pagnin E et al., the p66Shc mRNA was significantly increased in diabetes in contrast to controls 31. Our study failed to discover the correlation between p66Shc mRNA and age, weight and diabetes. The reason may be related to the small sample size and the exclusion of diabetes in this study.

A cross-sectional study with a small sample size is difficult to determine the causal link between the p66Shc gene expression in PBMC and the incidence rate of OSA is the main limitation of this study. Larger longitudinal research is needed to clarify the exact relationship between them.

## Conclusion

P66Shc mRNA and protein expression levels were significantly elevated in PBMCs of OSA patients and p66Shc mRNA levels were positively correlated with plasma concentrations of the oxidative stress indicators 3-NT, oxLDL, AOPP. AHI, MSaO_2_ and CT90 were independently associated with p66Shc mRNA levels. This suggests that oxidative stress regulator p66Shc may play a role in the pathophysiology of OSA and might serve as a potential biomarker for this disease.

## Figures and Tables

**Table 1:** Clinic characteristics of the subjects.

Variables	Control (n=19)	OSA (n=54)	*p* value
**Demographics**			
Age (years)	46.44±9.27	46.30±11.57	0.831
Sex (male, %)	12(63.2)	40(74.10)	0.286
BMI (kg/m^2^)	23.50(26.95, 20.20)	27.10 (25.00, 28.90)	0.008
SBP (mmHg)	132.83±14.17	133.49±14.94	0.885
DBP (mmHg)	82.39±9.27	87.24±11.25	0.147
Current smoker n (%)	3 (15.8)	16 (29.6)	0.237
Current drinker n (%)	3 (15.8)	16 (29.6)	0.237
**Sleep parameters**			
AHI (events/hour)	2.02±1.05	35.03±22.31	<0.001
LSaO_2_ (%)	83.68±11.15	71.82±15.69	0.003
MSaO_2_ (%)	96.14±1.26	93.35±2.98	<0.001
CT90 (%)	0.00(0.00, 0.80)	16.05(3.87, 51.4)	<0.001
ODI (events/hour)	3.26±3.04	41.39±26.79	<0.001
ESS	0.58±0.61	8.15±6.43	<0.001
**Clinical and biological parameters**			
WBC (10^9^/L)	5.29 (4.39, 7.57)	6.46 (4.97, 8.31)	0.049
LYM (10^9^/L)	1.32 (1.16, 2.00)	1.88 (1.50, 2.30)	0.006
MNC (10^9^/L)	0.29 (0.26, 0.38)	0.38(0.30, 0.48)	0.024
TG (mmol/L)	1.23 (0.80, 2.37)	1.88(1.56, 2.42)	0.027
TC (mmol/L)	4.28 (3.51, 5.05)	4.73(4.07, 5.20)	0.424
HDL-C (mmol/L)	1.22 (0.83, 1.39)	1.06(0.89, 1.34)	0.995
LDL-C (mmol/L)	2.56 (2.24, 3.29)	2.87(2.49, 3.57)	0.240
TP (g/L)	66.46±5.04	67.30±9.34	0.713
ALB (g/L)	40.30±2.75	41.18±4.91	0.465
GLO (g/L)	26.16±3.91	26.18±4.95	0.991
ALT (U/L)	19.66±6.98	27.36±13.41	0.039
AST (U/L)	16.70±5.83	21.24±7.01	0.025
TBIL (μmol/L)	7.88±2.84	9.17±2.83	0.133
DBIL (μmol/L)	3.41±1.75	3.09±0.94	0.365
TBA (μmol/L)	3.30(2.20, 4.20)	4.41(2.60, 5.30)	0.197
BUN (mmol/L)	4.89±1.29	5.04±1.42	0.686
CRE (μmol/L)	71.14±18.33	73.36±18.28	0.651
UA (μmol/L)	335.60(267.20, 360.30)	352.15(305.50, 396.32)	0.098
GLU (mmol/L)	4.79±0.66	5.12±0.92	0.175
**Oxidative stress marker**			
3-NT (ng/mL)	67.01±26.75	92.00±49.35	0.040
oxLDL (pg/mL)	2074.57±636.74	2545.99±867.07	0.033
AOPP (μmol/L)	217.81±127.25	336.83±172.18	0.007
p66Shc mRNA (AU)	0.84±0.44	1.63±1.51	0.029

Notes: Data are expressed as the means ± standard deviation for normally distributed data, median (interquartile range) for nonnormally distributed data, or number (n, %) for categorical variables. Abbreviations: BMI, body mass index; SBP, systolic pressure; DBP, diastolic pressure; AHI, apnea-hypopnea index; LSaO_2_, lowest oxygen saturation; MSaO_2_, mean oxygen saturation; CT90, percentage of total sleep time with SaO2 below 90%; ODI, oxygen desaturation index; ESS, epworth sleepiness scale; WBC, white blood cell; LYM, lymphocyte; MNC, monocyte; TG, triglyceride; TC, total cholesterol; HDL-C, high density lipoprotein cholesterol; LDL-C, low density lipoprotein cholesterol; TP, total protein; ALB, albumin; GLO, globulin; ALT, alanine transaminase; AST, aspartate aminotransferase; TBIL, total bilirubin; DBIL, direct bilirubin; TBA, total bile acid; BUN, blood urea nitrogen; CRE, creatinine; UA, uric acid; GLU, glucose; 3-NT, 3-nitrotyrosine; oxLDL, oxidized low density lipoprotein; AOPP, advanced oxidation protein products; AU, arbitrary units.

**Table 2:** Clinical variables in the control and OSA subgroups.

Parameters	Control (n=19)	Mild OSA (n=12)	Moderate OSA (n=15)	Severe OSA (n=27)
Age (years)	46.44±9.27	44.64±10.28	47.47±12.81	46.33±11.69
Sex (male, %)	63.2	58.30	86.70	77.80
BMI (kg/m^2^)	23.50(26.95, 20.20)	25.00(22.74, 32.15)	26.40(24.25, 27.20)	28.20(26.93, 29.58)^ *^
SBP (mmHg)	132.83±14.17	127.50±10.50	137.00±12.81	132.15±17.56
DBP (mmHg)	82.39±9.27	85.70±7.36	89.43±5.91	87.96±6.47
Current smoker n (%)	3 (15.8)	2 (16.7)	4 (26.7)	10 (37.0)
Current drinker n (%)	3 (15.8)	2 (16.7)	5 (33.3)	9 (33.3)
AHI (events/hour)	2.02±1.05	10.11±3.49^*^	22.17±2.77^*#^	52.33±17.83^*#&^
LSaO_2_ (%)	83.68±11.15	86.50±4.91	78.13±9.49	61.79±14.72^*#&^
MSaO_2_ (%)	96.14±1.26	95.98±1.09	94.63±1.40	91.48±2.98^*#&^
CT90 (%)	0.00(0.00, 0.80)	1.35(0.10, 3.58)	6.00(3.50, 17.20)^ *#^	49.9(20.80, 78.8)^ *#&^
ODI (events/hour)	3.26±3.04	10.25±6.29	30.60±9.15^*#^	60.19±23.09^*#&^
ESS	0.58±0.61	0.67±0.89	7.47±5.91^*#^	11.85±4.98^*#&^
3-NT (ng/mL)	67.01±26.75	76.09±28.86	78.00±41.99	105.16±57.90^*#&^
oxLDL (pg/mL)	2074.57±636.74	2233.36±447.03	2311.55±879.12	2815.19±937.96^*#^
AOPP (μmol/L)	217.81±127.25	239.01±172.57	281.87±151.87	410.84±153.52^*#&^
p66Shc mRNA (AU)	0.84±0.44	0.95±0.78	1.13±0.78	2.21±1.84^*#&^

Notes: Data are expressed as the means ± standard deviation for normally distributed data, median (interquartile range) for nonnormally distributed data, or number (n, %) for categorical variables. Abbreviations: BMI, body mass index; SBP, systolic pressure; DBP, diastolic pressure; AHI, apnea-hypopnea index; LSaO_2_, lowest oxygen saturation; MSaO_2_, mean oxygen saturation; CT90, percentage of total sleep time with SaO2 below 90%; ODI, oxygen desaturation index; ESS, epworth sleepiness scale; 3-NT, 3-nitrotyrosine; oxLDL, oxidized low density lipoprotein; AOPP, advanced oxidation protein products; AU, arbitrary units. *p < 0.05, compared with the control group. ^#^ *p* < 0.05, compared with the mild OSA group. ^&^* p* < 0.05, compared with the moderate OSA group.

**Table 3:** Association between levels of p66Shc mRNA and clinical variables in univariate and multivariate linear regression analyses.

Variables	Univariate analyses		Multivariate anlysis (R^2^=0.606, *p*< 0.001)	
	Beta (95%CI)	*p* value	Beta (95%CI)	*p* value
Age (years)	-0.015(-0.045,0.015)	0.509	-0.015(-0.044, 0.013)	0.263
Sex (male, %)	-0.066(-0.785,0.652)	0.406	0.105(-0.524, 0.734)	0.740
BMI (kg/m^2^)	0.082(-0.006,0.171)	0.066	-0.029(-0.111, 0.053)	0.479
AHI (events/hour)	0.033(0.022,0.044)	**<0.001**	0.067(0.031, 0.104)	**<0.001**
LSaO_2_ (%)	-0.048 (-0.066, -0.031)	**<0.001**	-0.008(-0.037, 0.021)	0.653
MSaO_2_ (%)	-0.170(-0.273, -0.067)	**0.002**	0.264(0.100, 0.428)	**0.002**
CT90 (%)	0.024(0.017, 0.031)	**<0.001**	0.020(0.008, 0.032)	**0.002**
ODI (events/hour)	0.024(0.014, 0.034)	**<0.001**	-0.024(-0.050, 0.002)	0.067
ESS	0.089(0.043, 0.143)	**<0.001**	-0.015(-0.082, 0.053)	0.668
WBC	-0.002(-0.137, 0.132)	0.974		
LYM (10^9^/L)	0.369(0.028,0.709)	**0.034**	-0.091(-0.410, 0.228)	0.571
MNC (10^9^/L)	-0.427(-1.760, 0.906)	0.525		
TG (mmol/L)	0.096(-0.270, 0.461)	0.603		
ALT (U/L)	0.000(-0.027,0.026)	0.974		
AST (U/L)	0.002(-0.046,0.051)	0.926		

Note: Multivariate linear regression model includes age, sex, BMI and other variables with *p*<0.05 in univariate model analyses. Abbreviations: CI, confidence interval; BMI, body mass index; SBP, systolic pressure; DBP, diastolic pressure; AHI, apnea-hypopnea index; LSaO_2_, lowest oxygen saturation; MSaO_2_, mean oxygen saturation; CT90, percentage of total sleep time with SaO2 below 90%; ODI, oxygen desaturation index; ESS, epworth sleepiness scale; LYM, lymphocyte; MNC, monocyte; TG, triglyceride; ALT, alanine transaminase; AST, aspartate aminotransferase; UA, uric acid;
